# *CHEK2* Germline Variants in Cancer Predisposition: Whole Genome Sequencing Results

**DOI:** 10.3390/ijms27177602

**Published:** 2026-08-25

**Authors:** Marina V. Nemtsova, Maria V. Makarova, Anastasiia M. Danishevich, Maria M. Byakhova, Olesya S. Mishina, Alevtina E. Kiseleva, Maxim S. Belenikin, Anastasia A. Krinitsina, Olesya V. Sagaydak, Anna B. Semenova, Natalia A. Bodunova, Igor E. Khatkov, Irina A. Demidova, Aleksey S. Tsukanov, Vsevolod N. Galkin, Saida M. Gadzhyeva

**Affiliations:** 1 Evogen LLC, Fotiyevoy St., 6, b.1, 119333 Moscow, Russia; nemtsova_m_v@mail.ru (M.V.N.); mishina@evogenlab.ru (O.S.M.); belenikin@evogenlab.ru (M.S.B.); krinitsina@evogenlab.ru (A.A.K.); sagaydak@evogenlab.ru (O.V.S.); 2 Research Centre for Medical Genetics, Moskvorechye St., 1, 115522 Moscow, Russia; 3 Federal State Autonomous Educational Institution of Higher Education I.M. Sechenov of the Ministry of Health of Russian Federation, Trubetskaya St., 8 p. 2, 119991 Moscow, Russia; kis-alevtina@yandex.ru; 4 Federal State Budgetary Institution Russian Scientific Center of Roentgenoradiology of the Ministry of Health of the Russian Federation, Profsoyuznaya St., 86, 117997 Moscow, Russia; 5 Moscow Clinical Scientific Centre Named After A.S. Loginov, Novogireevskaya St., 1 p. 1, 111123 Moscow, Russia; danishevicham@ya.ru (A.M.D.); n.bodunova@mknc.ru (N.A.B.); i.hatkov@mknc.ru (I.E.K.); 6 Moscow City Clinical Hospital Named After S.S. Yudin, Kolomensky pr., 4, 115446 Moscow, Russia; biakhovamm@mail.ru (M.M.B.); asemenova81@mail.ru (A.B.S.);; 7 Moscow City Oncology Hospital No. 62, Bolshoy Boulevard, 67, Skolkovo Innovation Center, 121205 Moscow, Russia; dema-80@yandex.ru; 8 Ryzhikh National Medical Research Centre for Coloproctology, Salama Adil Str., 2, 123423 Moscow, Russia; tsukanov81@rambler.ru; 9 Moscow Healthcare Department, Oruzheyniy per., 43, 127006 Moscow, Russia

**Keywords:** hereditary cancer syndromes (HCS), hereditary breast cancer, cancer risk, whole genome sequencing (WGS), *CHEK2*, c.1100del, c.444+1G>A, I157T, c.470T>C

## Abstract

While pathogenic germline *CHEK2* variants are known to increase cancer risk, there is currently insufficient evidence regarding the precise risk of developing malignant neoplasms associated with specific missense variants or variants of uncertain significance. As a result, no clear clinical guidelines exist regarding consultation, monitoring and specific treatment options for those patients. For the first time in Russia, clinical data and whole-genome sequencing (WGS) results were analyzed for 3150 patients with cancer and suspected hereditary cancer syndromes (HCS) and 5163 healthy individuals. This dataset formed the basis for assessing the role of germline *CHEK2* variants in the development of different cancer types. The chromosomal coordinates and coding sequence coordinates are given in accordance with the GRCh38 (hg38) genome assembly and the NM_007194.4 transcript. Pathogenic (P) and likely pathogenic (LP) variants of *CHEK2* significantly increased the risk of breast cancer (OR = 2.015 [95% CI: 1.27–3.21]; *p* = 0.0031), but the association with colorectal cancer was not statistically significant (OR = 1.354 [95% CI: 0.42–4.42]; *p* = 0.616). A moderate increase in cancer risk was identified for the c.1100del variant (OR = 2.263 [95% CI: 1.19–4.32]; *p* = 0.0132) and for the common P/LP variants c.1100del, c.444+1G>A and c.433C>T (OR = 2.219 [95% CI: 1.40–3.51]; *p* = 0.0007). Notably, our study confirmed that *CHEK2* c.470T>C (p.Ile157Thr) is the most common variant in the patient group, identified in 3.8% of cases (120/3150), compared with 3.0% in the control group (155/5163). Although the association between the most common *CHEK2* variant c.470T>C and cancer risk reached nominal statistical significance (OR = 1.279 [95% CI: 1.00–1.63]; *p* = 0.0463), the effect size was minimal, suggesting that the contribution of this variant to hereditary cancer risk in the Russian population is modest. Additional studies are required before this variant can be definitively excluded from clinical interpretation.

## 1. Introduction

The *CHEK2* gene, located on chromosome 22 (q12.1), encodes the effector checkpoint kinase 2 (CHK2), a key component of the ATM–CHK2–p53 pathway involved in the cellular response to DNA double-strand breaks (DSBs). In this pathway the ATM kinase phosphorylates CHK2, promoting its dimerization and activation. Once activated, CHK2 phosphorylates several downstream targets, including TP53, BRCA1 and other transcription factors that regulate cell-cycle arrest, apoptosis and cellular aging [1]. Both CHK2 and ATM phosphorylate BRCA1, which acts as an adaptor protein for the recruitment of BRCA2. BRCA2 contains a C-terminal domain including the TR2 region and eight conserved BRC repeats that serve as binding sites for RAD51. RAD51 forms nucleoprotein filaments that facilitate strand invasion into homologous DNA, playing a critical role in homologous recombination repair (HRR). It has been shown that pathogenic variants in *CHEK2* can disrupt these processes, leading to chromosomal instability in tumor cells [2].

Germline pathogenic variants in the *CHEK2* gene are shown to be associated with the predisposition to several tumor types, with breast cancer being the most extensively studied [3,4]. The potential association between germline *CHEK2* variants and an increased risk of colorectal, pancreatic, kidney, and prostate cancers is now being actively investigated [5]. Although a link between *CHEK2* pathogenic variants and Li–Fraumeni syndrome was initially proposed in 1999, subsequent studies disproved this association [6].

Today, *CHEK2* is included in next-generation sequencing (NGS) diagnostic panels for various hereditary cancer syndromes (HCS) and is considered one of the genes with a relatively high frequency of germline variants [7]. However, the prevalence and penetrance of specific variants vary substantially between different ethnic and geographic populations, underscoring the importance of studying *CHEK2* variants in representative patient and control cohorts in Russia.

Although CHK2 plays a key role in cell-cycle regulation following DNA damage, most *CHEK2*-associated tumors do not exhibit clear defects in homologous recombination repair (HRR), unlike *BRCA1/2*-associated tumors, and therefore may not respond to PARP-inhibitor [8]. Due to insufficient evidence supporting a markedly increased cancer risk, no standardized guidelines currently exist for genetic testing, clinical monitoring, or targeted treatment of individuals with germline *CHEK2* variants [9]. Several well-characterized pathogenic variants in the *CHEK2* gene have traditionally been linked to an elevated cancer risk, so the identification and analysis of other germline *CHEK2* variants in oncology patients requires further investigation.

The objective of the study was to characterize the frequency and spectrum of germline *CHEK2* variants in Russian patients with suspected hereditary cancer syndrome (HCS) identified by whole-genome sequencing, and to assess their association with an increased risk of malignant neoplasms.

## 2. Results

### 2.1. Frequency and Spectrum of CHEK2 Germline Variants Identified in Oncological Patients with Suspected Hereditary Cancer Syndromes and in a Control Group Without Neoplasms

Germline pathogenic (P) and likely pathogenic (LP) variants in the *CHEK2* gene were identified in 43 probands in the patient group (PG). The median age of P/LP carriers was 46.5 years, and 42 of the 43 carriers (97.7%) were female. The most common primary tumor among *CHEK2* carriers was breast cancer (35/43, 81.4%). The distribution by sex and diagnosis is explained by the majority of breast cancer patients included in the study, i.e., 2441 (77.5%). An amount of 42 patients had one variant and only one patient had two variants. In total, 191 *CHEK2* variants (19 different variants) were detected, including 44 P/LP (23%) and 147 variants of uncertain significance (VUS; 77%), yielding a P/LP-to-VUS ratio of 1:3.

Among pathogenic variants, the most recurrent variant was c.1100del (p.Thr367Metfs*15), accounting for 50% (22/44) of all P/LP. The second most frequent variant was the splice-site variant c.444+1G>A, detected in 27.3% (12/44). The distribution of pathogenic variant types was as follows: frameshift variants in 54.5% (24/44), missense variants in 18.2% (8/44) and splicing variants in 27.3% (12/44). The most prevalent VUS was the missense variant c.470T>C (p.Ile157Thr), which was detected in 81.6% (120/147). All identified *CHEK2* variants are summarized in Table 1.

The distribution of identified *CHEK2* variants across different cancer types is summarized in Table 2.

In the control group (CG), 192 *CHEK2* heterozygous variants were identified, of which 37 were classified as P/LP and 155 as VUS (c.470T>C). Other VUS were not considered in this group. The most prevalent PV was c.1100del, accounting for 16/37 of all P/LP variants. The splice-site variant c.444+1G>A was the second most frequently detected pathogenic variant, representing 12/37 of P/LP cases. The distribution of *CHEK2* variants identified in the control group is presented in Table 3.

Whole-genome sequencing (WGS) of both the patient group (PG) and the control group (CG) identified a previously reported structural variant: The deletion of *CHEK2* exons 9–10 (chr22:g.28696571–28701965del). The deletion was detected in 0.83% of individuals in the PG (26/3150) and in 0.62% of individuals in the CG (32/5163).

### 2.2. Risk Assessment of Malignant Neoplasms with Germline CHEK2 Variants

We evaluated the association between germline *CHEK2* variants and the risk of malignant neoplasms (MN) in the patient group (PG) with suspected hereditary cancer syndromes. Cancer risk analysis was conducted in three groups: breast cancer (BC), colorectal cancer (CRC) and ovarian cancer (OC). OR was calculated relative to all *CHEK2* variants (OR-1) and to only P/LP variants (OR-2). The complete results of the association analyses are presented in Table 4.

An association with cancer risk was found for the total number of *CHEK2* variants in patients with BC and CRC: OR-1 = 1.756 ([95% CI: 1.41–2.18], *p* < 0.0001) and OR-1 = 1.881 ([95% CI: 1.18–3.00], *p* = 0.0078) accordingly. When the analysis was restricted to P/LP variants, the OR for BC increased (OR-2 = 2.015 [95% CI: 1.27–3.21]; *p* = 0.0031). In contrast, the association with CRC was no longer statistically significant (OR-2 = 1.354 [95% CI: 0.42–4.42], *p* = 0.616).

Next, we evaluated the association with cancer risk for the common P/LP *CHEK2* variants: c.1100del, c.444+1G>A, and c.433C>T.

A moderate increase in cancer risk was observed for carriers of the c.1100del variant (OR = 2.263 [95% CI: 1.186–4.315]; *p* = 0.0132) and for the common P/LP *CHEK2* variants (OR = 2.219 [95% CI: 1.401–3.514]; *p* = 0.0007). When P/LP variants were analyzed together with the c.470T>C, the estimated risk for the patient group decreased substantially (OR = 1.671 [95% CI: 1.361–2.052]; *p* < 0.0001). Analysis restricted to the c.470T>C variant alone showed only a minimal, borderline significant increase in cancer risk (OR = 1.279 [95% CI: 1.00–1.63]; *p* = 0.0463). No association between the *CHEK2* structural variant chr22:g.28696571-28701965del and increased risk of malignant neoplasm development was observed in the patient group (OR = 1.335 [95% CI: 0.79–2.24]; *p* = 0.276). All risk estimates are summarized in Table 5.

At present, the association between germline *CHEK2* variants and breast cancer is the most extensively studied. However, data are gradually accumulating for other tumor types across diverse ethnic groups and patient cohorts. Currently, there is insufficient evidence to reliably establish disease risk associations between germline *CHEK2* variants and colorectal, renal, prostate, or pancreatic cancers. In this study, we also identified a 67-year-old male patient carrying two pathogenic *CHEK2* variants (c.1100del and c.444+1G>A in *trans*), who presented with an atypical phenotype of a hereditary cancer syndrome. His family history included a paternal grandfather with a malignant neoplasm of unspecified site, a father who died of accidental causes at age 50, and a mother diagnosed with breast cancer at age 67. Segregation analysis of the proband’s two children—both asymptomatic carriers of c.1100del—confirmed that c.1100del and c.444+1G>A are located on different alleles (in *trans*), establishing compound heterozygosity for two pathogenic loss-of-function *CHEK2* variants. The proband developed five metachronous primary tumors over a 20-year period: urothelial carcinoma of the bladder (pT1N0M0, 2003; with recurrences in 2005 and 2007) treated by transurethral resection; thyroid cancer and paraganglioma of the right neck (2006) treated by subtotal thyroidectomy and right cervical lymphadenectomy; renal clear cell carcinoma of the left kidney (pT1aN0M0, stage I, 2019) treated by laparoscopic partial nephrectomy; urothelial carcinoma of the right kidney (cT2N0M0, stage II, 2019) treated by laser ablation and with chemotherapy (gemcitabine + cisplatin, 2021–2022); and non-small cell lung cancer of the right upper lobe (cT4N0M0, stage IIIa, 2022) treated by right upper lobectomy and with chemotherapy (paclitaxel). This case suggests that biallelic pathogenic *CHEK2* variants may predispose male carriers to a broader spectrum of non-breast malignancies, potentially contributing to underdiagnosis in clinical practice.

## 3. Discussion

The *CHEK2* gene is characterized by a high mutational frequency in patients with cancer, particularly in Europeans, and has been reported as one of the most mutated cancer genes after *BRCA1* and *BRCA2*, especially among patients with breast cancer [7]. The relatively high prevalence of germline *CHEK2* variants in oncologic cohorts, together with the genes involved in key pathways regulating DNA damage response and oncogenesis, makes *CHEK2* an important candidate gene for explaining increased susceptibility to multiple tumor types. Numerous studies have investigated associations between germline *CHEK2* variants and cancer predisposition; however, clear and uniform clinical recommendations for monitoring and genetic testing of carriers of pathogenic/likely pathogenic *CHEK2* variants have remained limited. This challenge is further compounded by the routine inclusion of *CHEK2* in multigene cancer panels, which frequently leads to the identification of both pathogenic variants and variants of uncertain significance (VUS), complicating clinical interpretation. In 2023, the American College of Medical Genetics and Genomics (ACMG) published updated recommendations addressing the management of individuals with germline *CHEK2* variants, emphasizing the high population frequency of these variants and their incomplete penetrance [10]. In this context, our study provides additional evidence supporting the clinical relevance of pathogenic *CHEK2* variants. We demonstrated that carriers of P/LP germline *CHEK2* variants exhibit a moderate increase in breast cancer risk (OR = 2.015 [95% CI: 1.27–3.21]; *p* = 0.0031), as well as an increased overall cancer risk among patients with suspected hereditary cancer syndromes (OR = 2.219 [95% CI: 1.40–3.51]; *p* = 0.0007). These findings support the necessity of identifying and classifying pathogenic *CHEK2* variants in patients with suspected hereditary cancer syndromes, including Russian patients with hereditary breast cancer. In contrast, the association between the c.470T>C (p.Ile157Thr) variant and cancer risk reached only borderline statistical significance (OR = 1.279 [95% CI: 1.00–1.63]; *p* = 0.0463), with a minimal effect size. These findings suggest that the contribution of this variant to hereditary cancer risk in the Russian population is modest and may be lower than that of classical pathogenic *CHEK2* variants. These data should be taken into consideration during variant interpretation and genetic counseling of patients carrying the c.470T>C variant.

Our study confirmed that *CHEK2* c.470T>C (p.Ile157Thr) is the most common variant in both the patient group (3.8%, 120/3150) and the control group (3.0%, 155/5163). According to published data, the population frequency of heterozygous carriers of this variant is approximately 5% in Poland, Latvia, and Hungary and about 2–3% in the Czech Republic, Slovakia, and Germany [11]. Owing to its relatively high population prevalence, *CHEK2* c.470T>C is frequently detected in control groups in studies of various cancer types. Consequently, its contribution to breast cancer susceptibility has become increasingly controversial, and its role in breast cancer risk is currently being questioned. In a 2021 study, Novikova E.I. et al. demonstrated a high frequency of the *CHEK2* c.470T>C variant in a Russian cohort of patients with both benign breast diseases (BBDs) and malignant breast tumors, with frequencies of 4.7% and 3.8%, respectively. The absence of statistically significant differences between these groups led the authors to suggest that the c.470T>C variant is not associated with an increased risk of breast cancer [12].

In this study, we also described a clinical case of a patient with compound heterozygote for two pathogenic loss-of-function *CHEK2* variants and multiple primary tumors. The c.1100del variant is a well-characterized pathogenic variant that is relatively common in individuals of European ancestry and has been extensively studied across multiple cancer types. Meta-analyses have reported odds ratios ranging from 2 to 4 for the development of breast, colorectal, prostate, and other malignancies in carriers of this variant [13,14,15]. Functional studies have demonstrated loss of kinase activity and evidence of loss of heterozygosity in tumor tissue from carriers of the germline c.1100del variant. This frameshift variant introduces a premature termination codon, which is predicted to result in truncation of the protein or complete loss of protein expression due to nonsense-mediated mRNA decay, consistent with known pathogenic mechanisms. Based on these data and in accordance with ACMG criteria (PVS1, PP3, PP5), the c.1100del variant was classified as pathogenic.

The c.444+1G>A variant affects a canonical donor splice site. Functional studies in patient-derived cell lines have demonstrated activation of a cryptic splice site, resulting in the insertion of four base pairs into the mRNA and causing a frameshift [16]. This variant has been reported in numerous patients with breast cancer, including individuals with bilateral disease [17,18]. Additionally, c.444+1G>A has been associated with an increased risk of prostate, thyroid, and gastric cancers [19,20]. Based on available evidence and in accordance with ACMG criteria (PVS1, PP3, PP5, PM2, PS3), the c.444+1G>A variant was classified as pathogenic.

The segregation analysis was recommended for first-degree relatives. This analysis confirmed that the c.1100del and c.444+1G>A variants are located in *trans*. Consequently, the proband was identified as a compound heterozygote for two pathogenic loss-of-function *CHEK2* variants. Heterozygous carriage of a pathogenic *CHEK2* variant is associated with an increased risk of developing malignancies at multiple locations. In this clinical case, the presence of two pathogenic variants in *trans* supports the diagnosis of a *CHEK2*-associated hereditary cancer syndrome, underscoring the importance of early diagnosis and targeted testing to prevent or detect malignant neoplasms at an early stage.

Although the association between germline *CHEK2* variants and the development of urothelial carcinoma has not yet been conclusively established, several studies have reported frequent detection of germline *CHEK2* variants in families with hereditary urothelial carcinoma, alongside variants in *FANCM*, *MSH6*, *MSH2*, *BRCA2*, *ERCC3*, and *BRIP1* [21], as well as in patients with renal cancer [22]. At present, insufficient data are available regarding cancer risks in individuals with biallelic variants in the *CHEK2* gene, and existing observations predominantly concern patients with breast cancer [23].

In the clinical case presented in our study, a male patient carrying two pathogenic variants in *CHEK2* exhibited a heterogeneous tumor spectrum, including urothelial carcinoma, thyroid and renal cancers. Furthermore, Snežana Hinić et al. conducted a large cohort study of patients with biallelic *CHEK2* variants and suggested that men with biallelic germline pathogenic variants in *CHEK2* are more likely to develop cancer types other than breast cancer, and that tumor manifestation in men may occur at a later age when compared with women [24]. This may result in underdiagnosis of individuals with biallelic *CHEK2* pathogenic variants, as *CHEK2* testing is often limited to patients with breast cancer or individuals with a family history of breast cancer.

We are the first to evaluate the role of germline structural variants in the *CHEK2* gene in the development of tumors of various localizations using whole-genome sequencing of Russian oncological patients with suspected hereditary cancer syndromes, alongside a control group without neoplasms. We confirmed an association between increased cancer risk and certain pathogenic *CHEK2* variants in both the overall oncologic cohort and in patients with breast cancer, supporting the inclusion of *CHEK2* in multigene panel testing for Russian patients. The results obtained in this study may be used to inform the development of recommendations for the monitoring and genetic testing of patients carrying pathogenic variants in the *CHEK2* gene.

## 4. Materials and Methods

The study cohort consisted of two groups: 3150 patients with cancer and suspected hereditary cancer syndromes (patient group (PG)) and 5163 healthy individuals without evidence of malignancy (control group (CG)).

The patient group (PG) consisted of 3150 patients with suspected hereditary cancer syndromes (HCS) referred for genetic testing by five state budgetary healthcare institutions in Moscow: Moscow City Clinical Hospital, named after S.S. Yudin (formerly City Clinical Cancer Hospital No. 1); Moscow Clinical Scientific Centre, named after A.S. Loginov; Moscow City Oncology Hospital No. 62; Moscow Botkin Multidisciplinary Scientific-Clinical Center; and Moscow Multidisciplinary Clinical Center “Kommunarka”. Inclusion criteria comprised (1) a confirmed diagnosis of a malignant neoplasm; (2) age at diagnosis ≤ 60 years for breast, colorectal, or ovarian cancer, or any age with a documented family history of cancer; (3) bilateral or multifocal disease; or (4) a personal or family history meeting established criteria for genetic testing (e.g., NCCN/ESMO guidelines). All included patients had a histologically or cytologically confirmed cancer diagnosis. Healthy relatives without a cancer diagnosis were not included in the PG. The PG included patients with the following primary tumor types: breast cancer (BC; *n* = 2441, 77.5%), colorectal cancer (CRC; *n* = 310), ovarian cancer (OC; *n* = 191), pancreatic cancer (PC; *n* = 116), gastric cancer (GC; *n* = 45), endometrial cancer (EC; *n* = 39), and other tumors (*n* = 8). The cohort was predominantly female, consisting of 2925 women (92.9%) and 225 men (7.1%). The overall age range spanned from 18 to 88 years, with a mean age of 47.5 ± 9.9 years. When stratified by sex, women had a mean age of 47.2 ± 9.6 years (range 18–88 years), while men had a higher mean age of 52.3 ± 13.1 years (range 20–84 years).

The control group (CG) consisted of 5163 healthy Russian female individuals with ages ranging from 20 to 73 years (mean age 40.8 ± 9.5 years) who underwent whole-genome sequencing at Evogen LLC as part of population-based screening or clinical workup unrelated to hereditary cancer syndromes. No formal age-matching was applied. All control individuals were self-reported as free of malignancy at the time of sample collection.

Informed consent was obtained from all subjects involved in the study.

Whole-genome sequencing (WGS) was performed at the Evogen LLC using DNBSEQ-T7 and DNBSEQ-G400 next-generation sequencing platforms (MGI, Shenzhen, China) with a PCR-free enzymatic fragmentation library preparation protocol (MGI Easy FS PCR-Free DNA Library Prep Kit, MGI, China). Paired-end sequencing (2 × 150 bp) was conducted in accordance with the standard protocols, achieving an average sequencing depth of 30×. Variant calling and annotation were performed using bioinformatics analysis platforms EVA Pro (Evogen, Moscow, Russia) and Mega BOLT (MGI, China). During WGS data analysis, particular emphasis was placed on cancer-associated genes. Population allele frequencies of identified variants were assessed using the gnomAD database.

Validation of WGS-identified *CHEK2* variants in the patient group was performed by Sanger sequencing at Evogen LLC. Genomic DNA was extracted using the QIAamp DNA Blood Mini Kit (QIAGEN, Hilden, Germany) or MGI Easy Magnetic Beads (MGI, China) according to the standard protocols. PCR products were purified and sequenced using the BigDye Terminator v3.1 Cycle Sequencing Kit and an ABI 3500 Genetic Analyzer (Applied Biosystems, Carlsbad, CA, USA). Sequence data were analyzed using Variant Reporter Software v3.0 (Applied Biosystems, USA).

The chromosomal coordinates and coding sequence coordinates are given in accordance with the GRCh38 (hg38) genome assembly and the NM_007194.4 transcript.

**Figure 1:**
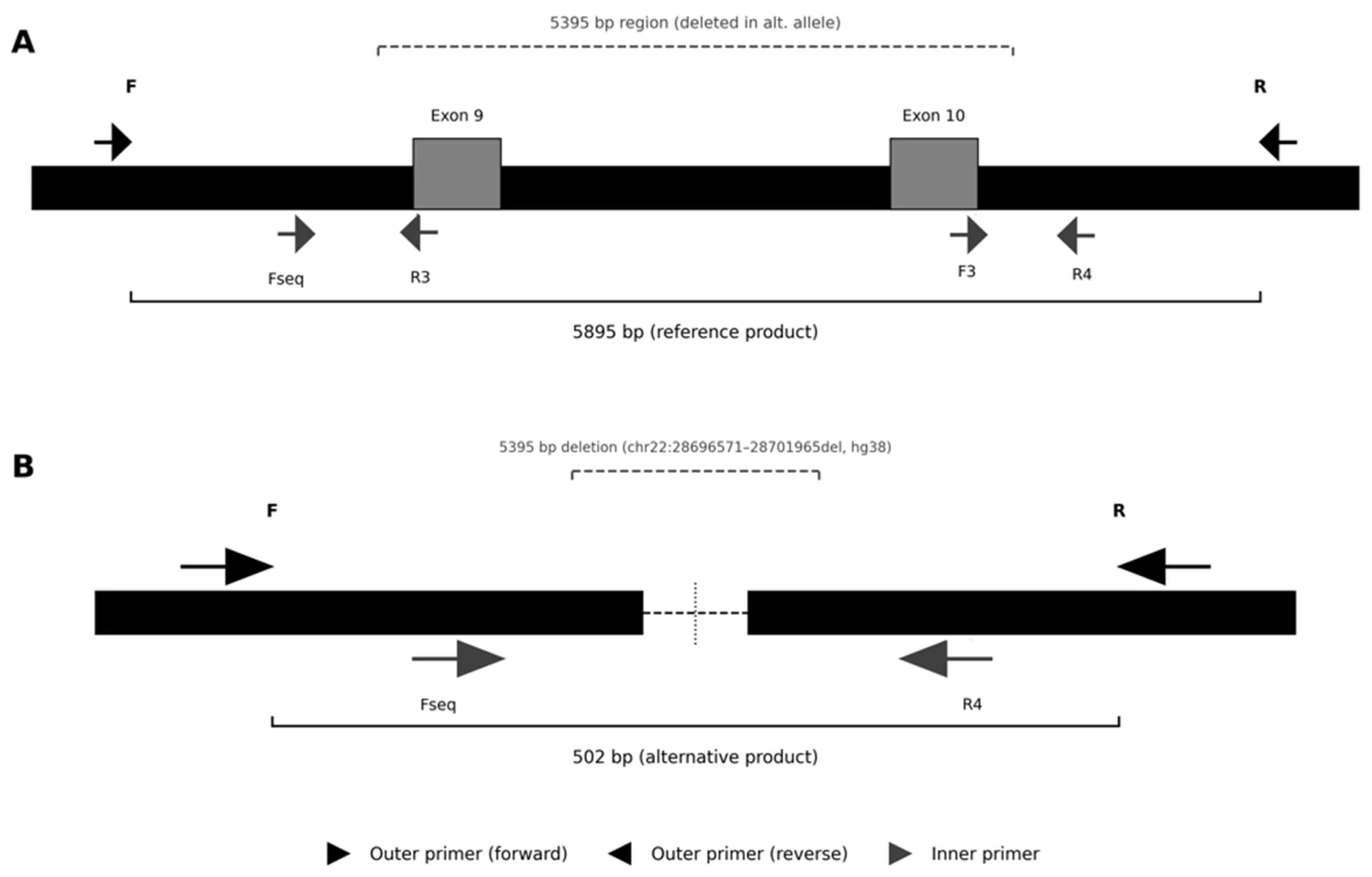
Schematic representation of the primer system designed for PCR-based detection and Sanger sequencing validation of the *CHEK2* exons 9–10 deletion (chr22:g.28696571–28701965del, hg38). (**A**) Reference allele: Exons 9 and 10 (filled gray rectangles) are intact within the 5395 bp region flanked by outer primers F (indicating forward) and R (indicating reverse), yielding a 5895 bp PCR product. Inner primers Fseq/R3 and F3/R4 are positioned near the left and right boundaries of the deleted region, respectively, for Sanger sequencing reactions. (**B**) Alternative allele: The 5395 bp region encompassing exons 9–10 is deleted and the flanking sequences are joined at the breakpoint junction; PCR with the outer primers F and R yields a shorter 502 bp product, while the inner primers Fseq and R4 enable Sanger sequencing across the breakpoint junction for precise coordinate refinement.

To confirm the presence of the *CHEK2* deletion (chr22:g.28696571–28701965del) and to refine its breakpoint coordinates, a custom primer system was designed. Outer primers F and R were positioned upstream and downstream of the predicted structural variant boundaries. PCR amplification with this primer pair was expected to yield two products: a reference allele product of 5895 bp (without deletion) and an alternative allele product of 502 bp (harboring the deletion). Four inner primers—Fseq, F3, R3, and R4—were designed for direct Sanger sequencing of the amplicons. Sanger sequencing was performed using three complementary approaches to characterize the deletion junction and refine the breakpoint coordinates. First, the alternative allele amplification product (502 bp) was fully sequenced to identify the breakpoint junction. Second, the 5′ and 3′ portions of the long reference allele product (5895 bp) were sequenced with the expectation that the resulting chromatograms would contain part of the deleted region, thereby confirming the reference-state sequence flanking the breakpoints. Third, a mixture of reference and alternative amplification products was sequenced, with the expectation that chromatogram peak shifts corresponding to the overlap of the two allelic states would be observed at the deletion boundaries. All sequencing reactions were carried out with bidirectional reads to ensure independent confirmation of each breakpoint. The structural variant coordinates were refined by aligning the Sanger sequencing reads against the GRCh38/hg38 reference genome assembly using the UCSC Genome Browser (https://genome.ucsc.edu, accessed on 24 July 2026). The deletion was confirmed to span 5395 bp, with the first nucleotide of the deleted region at chr22:g.28696571 and the last nucleotide at chr22:g.28701965. The final validated coordinates of the structural variant were designated as chr22:g.28696571–28701965del. A schematic overview of the primer arrangement on both the reference and alternative alleles is provided in Figure 1.

Statistical analysis. Odds ratios (OR) with 95% confidence intervals (CI) were calculated to assess the association between germline *CHEK2* variants and cancer risk. The analysis focused on a limited number of pre-specified *CHEK2* variants selected on the basis of prior biological evidence and published literature, making formal multiple testing correction less critical for the principal conclusions. While some comparisons are hierarchically related—individual variants are subsets of combined P/LP and total variant analyses—the key findings (*p* < 0.001 for combined P/LP variants; OR = 2.219) are robust and insensitive to the choice of correction method. The control group consisted entirely of female individuals (*n* = 5163), precluding sex-adjusted or sex-stratified analysis due to the absence of male controls. The marginal association observed for c.470T>C (p.Ile157Thr; OR = 1.279, [95% CI: 1.00–1.63]; *p* = 0.0463) is addressed in the Discussion through cautious interpretive language, acknowledging its modest effect size and the need for additional studies before any clinical exclusion of this variant.

## 5. Conclusions

This study presents the spectrum of *CHEK2* gene variants identified by whole-genome sequencing in patients with suspected hereditary cancer syndromes. The highest frequency of pathogenic and likely pathogenic *CHEK2* variants was observed among patients with breast cancer. Overall, our results highlight the importance of distinguishing pathogenic *CHEK2* variants from frequent low-penetrance or uncertain variants to optimize risk stratification, monitoring strategies and genetic testing for patients with suspected hereditary cancer syndromes. Three *CHEK2* variants were identified as the most frequent in cancer patients: c.1100del (p.Thr367MetfsTer15), c.444+1G>A, and c.433C>T (p.Arg145Trp).

In addition, the article also presents data from both Russian and international studies, indicating that the clinical significance of the *CHEK2* c.470T>C (p.Ile157Thr) variant in breast cancer susceptibility is limited, with only a modest and borderline significant association with cancer risk observed in our cohort. Given its high population frequency, the authors suggest that the clinical utility of including *CHEK2* c.470T>C (p.Ile157Thr) in diagnostic panels may be limited; however, definitive exclusion from clinical risk assessment requires further investigation.

## Figures and Tables

**Table 1:** The spectrum of *CHEK2* variants identified in the patient group (PG).

№	Variant	rs ID	Number of Variants	Allele Frequency (%) *	Clinical Significance
1	c.1100del (p.Thr367MetfsTer15)	rs555607708	22	0.172	P
2	c.444+1G>A	rs121908698	12	0.0085	P/LP
3	c.433C>T (p.Arg145Trp)	rs137853007	8	0.0046	P/LP
4	c.1263del (p.Ser422ValfsTer15)	rs587780174	2	0.0059	P
Total P+LP			44		
5	c.470T>C (p.Ile157Thr)	rs17879961	120	0.404	VUS
6	c.541C>T (p.Arg181Cys)	rs137853010	4	0.0059	VUS
7	c.1270T>C (p.Tyr424His)	rs139366548	4	0.0191	VUS
8	c.1283C>T (p.Ser428Phe)	rs137853011	3	0.025	VUS
9	c.538C>T (p.Arg180Cys)	rs77130927	3	0.0618	VUS
10	c.480A>G (p.Ile160Met)	rs575910805	2	0.0033	VUS
11	c.1312G>T (p.Asp438Tyr)	rs200050883	2	0.0374	VUS
12	c.1067C>T (p.Ser356Leu)	rs121908703	2	0.0007	VUS
13	c.972C>G (p.Cys324Trp)	rs1060502712	1	-	VUS
14	c.542G>A (p.Arg181His)	rs121908701	1	0.0059	VUS
15	c.190G>A (p.Glu64Lys)	rs141568342	1	0.0145	VUS
16	c.751A>T (p.Ile251Phe)	rs587780189	1	0.0079	VUS
17	c.980A>G (p.Tyr327Cys)	rs587780194	1	0.002	VUS
18	c.1091T>C (p.Ile364Thr)	rs774179198	1	-	VUS
19	c.1542G>T (p.Gln514His)	rs747797219	1	-	VUS
Total VUS			147		
Total			191		

* allele frequency according gnomAD genomes v.4.1.0 data; P—pathogenic, LP—likely pathogenic; VUS—variant of uncertain significance.

**Table 2:** Distribution of the germinal P/LP *CHEK2* gene variants in patients with different cancer types.

№	Primary Tumor	Number of Patients	Number of Variants
1	BC	2441	35
2	CRC	310	3
3	OC	191	3
4	PC	116	1
5	GC	45	-
6	EC	39	-
7	Other cancer types	8	2
Total		3150	44

BC—breast cancer, CRC—colorectal cancer, OC—ovarian cancer, PC—pancreatic cancer, GC—gastric cancer, EC—endometrial cancer.

**Table 3:** The spectrum of *CHEK2* variants identified in the control group.

№	Variant	rsID	Number of Variants	Clinical Significance
1	c.1100del (p.Thr367MetfsTer15)	rs555607708	16	P
2	c.444+1G>A	rs121908698	12	P/LP
3	c.433C>T (p.Arg145Trp)	rs137853007	4	P/LP
4	c.893_897del (p.Tyr298CysfsTer12)	rs1390889028	2	P
5	c.1368dup (p.Glu457ArgfsTer33)	rs730881700	1	P
6	c.319+2T>C	rs587782401	1	P/LP
7	c.319+2T>A	rs587782401	1	P/LP
Total P+LP			37	
8	c.470T>C (p.Ile157Thr)	rs17879961	155	VUS
Total			192	

P—pathogenic, LP—likely pathogenic, VUS—variant of uncertain significance.

**Table 4:** Association between germline *CHEK2* variants and the risk of breast cancer, colorectal cancer and ovarian cancer.

№	Primary Tumor	Number of Patients	*CHEK2* Variants Total	OR-1	*CHEK2* PV/LP	OR-2
1	BC	2441	155	1.756 [95% CI: 1.41–2.18], *p* < 0.0001	35	2.015 [95% CI: 1.27–3.21], *p* = 0.0031
2	CRC	310	21	1.881 [95% CI: 1.18–3.00], *p* = 0.0078	3	1.354 [95% CI: 0.42–4.42], *p* = 0.616
3	OC	191	11	1.582 [95% CI: 0.85–2.96], *p* = 0.151	3	2.211 [95% CI: 0.68–7.24], *p* = 0.190

BC—breast cancer, CRC—colorectal cancer, OC—ovarian cancer, OR-1—OR to all *CHEK2* variants, OR-2—OR to only P/LP variants.

**Table 5:** Risk associated with common germline *CHEK2* variants.

№	Genetic Variant	Clinical Significance	PG *n* = 3150	CG *n* = 5163	OR	*p* Value
1	c.1100del (p.Thr367MetfsTer15) rs555607708	P	22	16	2.263 [95% CI: 1.19–4.32]	0.0132
2	c.444+1G>A rs1219086	P/LP	12	12	1.642 [95% CI: 0.74–3.66]	0.226
3	c.433C>T (p.Arg145Trp) rs137853007	P/LP	8	4	3.284 [95% CI: 0.99–10.92]	0.0523
4	c.470T>C (p.Ile157Thr) rs17879961	VUS	120	155	1.279 [95% CI: 1.00–1.63]	0.0463
5	chr22:g.28696571-28701965del	P/LP	26	32	1.335 [95% CI: 0.79–2.24]	0.276
Three common P/LP SNVs		P+LP	42	32	2.219 [95% CI: 1.40–3.51]	0.0007
Total *CHEK2* detected SNVs		P+LP+ VUS	191	192	1.671 [95% CI: 1.36–2.05]	<0.0001

P—pathogenic variant, LP—likely pathogenic variant, VUS—variant of uncertain significant, PG—patient group, CG—control group, SNV—single nucleotide variant.

## Data Availability

The data analyzed in this study is not publicly available. Requests to 314 access the datasets should be directed to the following email: belenikin@evogenlab.ru.
